# Barriers and facilitators to the implementation of nurse’s role in primary care settings: an integrative review

**DOI:** 10.1186/s12912-021-00696-y

**Published:** 2021-09-16

**Authors:** Erica Busca, Alessia Savatteri, Tania Lorenza Calafato, Beatrice Mazzoleni, Michela Barisone, Alberto Dal Molin

**Affiliations:** 1 Department of Translational Medicine, University of Piemonte Orientale, Via P. Solaroli, 17, 28100 Novara, Italy; 2 Functional Oncology Department – Gastroenterology, Hospital “San Vincenzo” of Taormina, Contrada Sirina, 98039 Taormina, Italy; 3 Emergency-Urgency Department, Hospital “Sant’Elia” of Caltanissetta, Via Cusmano,1, 93100 Caltanissetta, Italy; 4 Humanitas University, Via Rita Levi Montalcini 4, Pieve Emanuele, 20090 Milan, Italy; 5 Department of Translational Medicine, University of Piemonte Orientale, Direzione delle Professioni Sanitarie - A.O.U. Maggiore della Carità di Novara, Via P. Solaroli, 17, 28100 Novara, Italy

**Keywords:** Registered nurse, Nurse practitioner, Advanced nurse practitioner, Family nurse practitioner, Family health nurse, Community health nurse, District nurse, Public health nurse, Primary care, Community care

## Abstract

**Background:**

The rapid evolution of the epidemiological picture and the recent SARS-COV-2 pandemic has expressed the vulnerabilities of health systems and focuses attention on the population’s needs.

The nurse’s figure in the care teams is universally identified; however, the implementation of the role within some care settings turns out to be complex and challenging. This integrative review aims to identify the barriers and facilitators in implementing the role of the nurse in primary care settings.

**Methods:**

An integrative review was conducted on the Medline and Cinahl databases until 9 June 2020. Qualitative, quantitative, and Mixed-method research studies were selected to identify studies related to the barriers and facilitators of the nurse’s role in nursing facilities’ primary care. For the extraction of the results, the Consolidating Framework for Research Implementation (CFIR) was used to identify the factors that influence implementation in health care.

**Results:**

Following the duplicates’ removal, the search identified 18,257 articles, of which 56 were relevant to the inclusion criteria; therefore, they were included in the summary.

The selected studies were conducted in thirteen countries, most from Oceania, Europe, North America, Latin America, and the Caribbean.

The barriers reported most frequently concern the nursing profession’s regulatory and regulatory aspects within the contexts of care, cultural and organizational aspects, training, and the transfer of specific skills, which were previously designated to doctors.

The facilitators are mainly linked to the nurse’s adaptability to the various contexts of care, recognizing the patient’s role, and the desire to develop multidisciplinary and effective working groups to respond to the health needs of the population in primary care contexts.

**Conclusion:**

This review highlighted the main barriers and facilitators in implementing the nurse’s role in primary care settings. These results offer useful elements for stakeholders to identify effective strategies in preparing programs and activities for implementing the nurse’s role, acting on the elements identified as barriers and favouring the aspects that emerge as facilitators.

**Supplementary Information:**

The online version contains supplementary material available at 10.1186/s12912-021-00696-y.

## Background

In recent years, the progressive epidemiological changes in large part due to the aging population, the increase in non-communicable diseases (NCDs), and the recent COVID-19 pandemic have necessarily led to a rethinking of the people’s needs for assistance, redefining the models of care for the most vulnerable age groups [1, 2].

NCDs, such as heart disease, stroke, cancer, diabetes, and chronic lung disease, have become the leading cause of disability and death worldwide [3]. In 2017, one in eight people was aged 60 years or older, and it is estimated that there will be one person over 60 for every six and five people by 2030 and 2050, respectively [4].

To counteract this emerging public health problem, the World Health Assembly of the World Health Organization (WHO) has launched an initiative named Decade of Healthy Aging 2020–2030 [5] aimed to promote autonomy among the elderly while designing new patient-focused care models and identifying long-term care needs. If no action is taken, health spending, tax burden, and health inequalities, especially in low and middle-income countries, are all expected to increase significantly in the nearby future [6]. Thus, there is a growing consensus among citizens that strengthening the resilience of national healthcare systems will help mitigate the impact of the epidemiological changes.

The recent COVID-19 pandemic has further increased the complexity of care and created an even greater demand for *chronic* care services carried out at the patient’s home [7, 8]. This has led to an in-depth reflection on current models of care, raising the important issue of what role nurses should play to help meet the increasingly complex healthcare needs of the community.

In most countries, one of the main reasons for developing and implementing the nurse’s role is to improve access to healthcare, especially in those settings where medical resources are scarce [9]. Another equally important reason for developing nursing nurses’ roles is that this process is critical to further promote the quality of care by providing support to chronic patients through on-site follow-up activities, thereby reducing hospital admissions and readmissions [10].

However, the implementation of nursing roles is not unique at an international level. There are, in fact, cultural, regulatory, and organizational factors specific to individual contexts that should be taken into account besides the nursing skill-mix level [11]. Thus, the epidemiological evolution we are witnessing requires the redefinition of the roles of the various professionals involved in primary care assistance aimed to enhance professional collaboration and, at the same time, redefine the nursing skills [12]. In particular, the heterogeneity of nursing contexts and roles at the international level calls for the need to define new strategies for implementing nursing roles in primary care settings [13].

In light of these considerations, the WHO guidelines have set the standards to achieve a sustainable primary healthcare system in line with the legislation, organization, and health priorities of each individual nation, prioritizing disease prevention and promoting health. By offering effective services in the field of prevention, promotion, treatment, rehabilitation, and palliative care, the ambitious goal of this initiative is that of fulfilling people’s health needs throughout their lives in a sustainable way [14]. Therefore, it is becoming increasingly clear how theoretical and clinical skills acquired by nurses through training and retraining will be key to the implementation of care roles and the improvement of health outcomes in primary care settings [15].

However, a large body of literature has pointed to several factors influencing the effectiveness of nurse’s role implementation in the primary care settings [13]. Thus, the purpose of this study was to identify the facilitators and barriers encountered during nurse’s role implementation from the stakeholders’ perspective (i.e., nurses, physicians, and patients).

## Methods

### Study design

The research question was addressed through an integrative review method that allows using original qualitative research and quantitative research on barriers to and facilitators of nurse’s role implementation in primary care settings [16]. This integrative review combines data from studies conducted using various designs and provides an in-depth analysis of this complex theme. The Preferred Reporting Items for Systematic Review and Meta-Analyses (PRISMA) was used [17].

### Search strategy

The search was performed using the two databases Medline and CINAHL, up to the 9th of June 2020. We developed search strategies for each database (Additional file 1). Search strategies consisted of keywords and controlled vocabulary terms (Table 1). We also scanned reference lists of all included studies and key references (i.e., relevant reviews). We limited our searches to English and Italian for feasibility reasons.

**Table 1:** Terms used in search strategies

MeSH terms*	Relevant key words**
Nurse practitioners	Nurse practitioner, advanced nurse practitioner
Nurses, Community Health	Family nurse practitioner, family health nurse, community health nurse, district nurse, public health nurse, rural nurse
Family Nurse Practitioners	
Nurses, Public Health	
Primary health care	Primary care, community care, community health care, district
Community Health Services	
Nurse’s Role	Nurse role

*MeSH terms were combined in three different searches using Boolean operators AND, and the search terms within each box were combined with OR

**Keywords were searched using truncation and phrase symbols when appropriate

### Eligibility criteria

We included primary studies that used qualitative or quantitative study designs and mixed methods approaches. We excluded case studies, editorials, commentaries, and reviews. We included studies that focused on stakeholders’ perceptions of how nurse’s role implementation is developed. Stakeholders include nurses, general practitioners, patients, and other individuals or professional categories directly or indirectly affected by nurse’s role implementation in primary care settings. We included any types of nurses working in primary care settings. Primary care was defined as follows: “*The provision of universally accessible, integrated person-centred, comprehensive health and community services provided by a team of professionals accountable for addressing a large majority of personal health needs. These services are delivered in a sustained partnership with patients and informal caregivers, in the context of family and community, and play a central role in the overall coordination and continuity of people’s care”* [18]*.*

We excluded studies focused on nurses or nursing practice concepts conducted in settings other than primary care (e.g., hospital emergency departments). Studies conducted in mixed settings were included if the results related to primary care could be clearly identified among the overall findings.

### Selection of studies

Two review authors independently scanned each title and abstract obtained from the electronic databases to determine if these fulfilled the inclusion criteria. Then, full-text publications of the selected studies were retrieved to confirm they met inclusion criteria. At all stages, we resolved any disagreements between the authors via discussion or, if required, by seeking a third reviewer’s opinion.

### Data extraction

We perform data extraction using the Consolidating Framework for Research Implementation (CFIR). The CFIR structure supports the exploration of essential factors encountered during implementation through formative evaluations [19] (Table 2). The framework emphasizes the multi-level influences on nurse’s role implementation, from external influencers to organizational and core implementation process components, and provides a pragmatic organization of constructs.

**Table 2:** Descriptions of CFIR domains

Domain	Definition
**Intervention characteristics**	The characteristics of the intervention being implemented include whether the intervention is perceived to be developed external or internal to the organization, there is evidence supporting its effectiveness, and its implementation will be advantageous to its alternatives. Other characteristics include how the intervention is presented, its adaptability, complexity and whether it can be tested on a smaller scale.
**Outer setting**	The external context of the organization includes patient needs and the ability to meet them, networks with other organizations, pressure to implement the intervention and external policies and incentives to adopt the intervention.
**Inner setting**	Features of the organization including its structural characteristics (such as size, age of the organization and division of labour), networks and communication (such as connections and information sharing between individuals, units and services), cultural norms and values, implementation climate, organizational capacity and readiness for change.
**Characteristics of individuals**	Staff knowledge and belief about the intervention, their ability to execute their respective aspects of the implementation, and their individual stage of change. Other characteristics include individual identification with the organization and other personal attributes.
**Process**	Active change process, the purpose of which is to promote uptake of the intervention by the organization. This is influenced by the level of planning prior to implementation, and engaging organization stakeholders through appointing implementation leaders and champions of the intervention. This includes the ability to execute the implementation of the intervention as planned and to continuously reflect on and evaluate the quality of implementation and intervention as it progresses.

We also extracted information on study characteristics (i.e., author, date of publication, country, aims, study design, study population, and study setting) and a description of the nurse’s role (i.e., training and details about any interventions delivered).

### Data synthesis

Three review authors read the selected studies and applied the CFIR framework, moving between the framework themes. Relevant data of each theme were extracted from all primary data sources. The review author, after discussing each emerging theme, definition, and boundaries, revised and compiled the CFIR framework in line with the emerging categories.

### Quality appraisal

Whittemore and Knafl (2005) state that assessing the quality of the included evidence is not essential in a supplementary review [16]. All studies meeting the inclusion criteria, regardless of their methodological quality, were retained in the review to examine all evidence of the factors that influenced the nursing role implementation in practice settings.

## Results

### Characteristic of the included studies

**Fig. 1:**
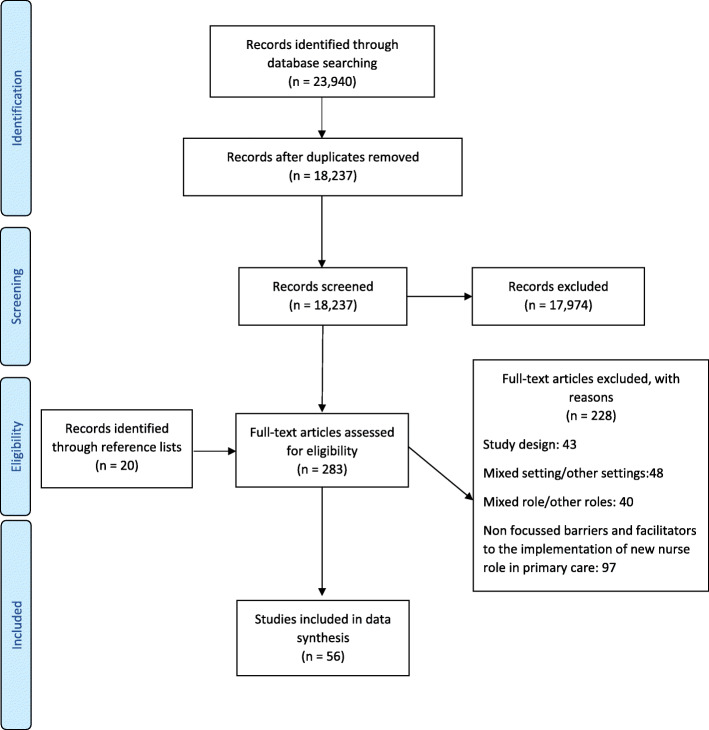
PRISMA flow diagram

We screened 18,257 records and considered 283 full texts for inclusion in this integrative review. Fifty-six papers met the inclusion criteria [20–75], and six papers [30, 45, 47, 49, 59, 61] derived from three unique studies (Fig. 1).

Studies were conducted across 13 countries: 9 studies in Oceania [26, 32, 35, 44, 60, 66, 68–70], one in Asia [36], 21 in Europe [20–22, 29, 33, 34, 41–43, 46, 51–54, 57, 58, 62, 65, 67, 73, 74], 24 in North America [23–25, 27, 28, 30, 31, 37–40, 45, 47–50, 55, 56, 59, 61, 64, 71, 72, 75], and one in Latin America and the Caribbean [63]. Thirty-six studies employed a qualitative design either descriptive [20, 22, 25, 29, 31, 36, 38, 41–44, 46, 48, 52–59, 61, 62, 64, 69, 71, 73], grounded theory [51, 70, 74], phenomenological approach [32, 40], or ethnographic research [26, 35, 39]. Fourteen studies used a quantitative design -cross sectional approach- [21, 24, 27, 28, 30, 34, 37, 45, 47, 49, 60, 63, 72, 75], while 6 used a mixed method [23, 33, 50, 65, 66, 68].

Participants included registered nurses, nurse practitioners, general practitioners, health leaders (chairpersons of health boards), managers, nursing leaders, key informants (e.g., university employees, Ministry of Health employees, policy makers), health and social care professionals, administrators, and patients (Additional file 2).

### Nursing role and tasks

A number of studies took into account nurse practitioners working in advanced roles (APN) [21, 23, 24, 26–31, 35, 37–42, 44–56, 59, 61–64, 66–69, 72, 74, 75] and registered nurses working in advanced practice levels or with specialist designations [20, 22, 25, 32, 33, 36, 42, 43, 57, 58, 60, 65, 70, 71, 73].

In these studies, the title “registered nurse” was often replaced by the following definitions: “community nurse”, “family health nurse”, “public health nurse”, “mental health nurse”, “community matron”, “mental health nurse of community”, or “district nurse”.

A number of studies specified nurses’ qualifications, ranging from bachelor’s degree to post-graduate qualification attainment (e.g., master’s degree, doctorate in nursing) [24–31, 35, 36, 38–42, 44, 48, 49, 51–53, 56, 63, 64, 66–69, 72, 74, 75].

**Fig. 2:**
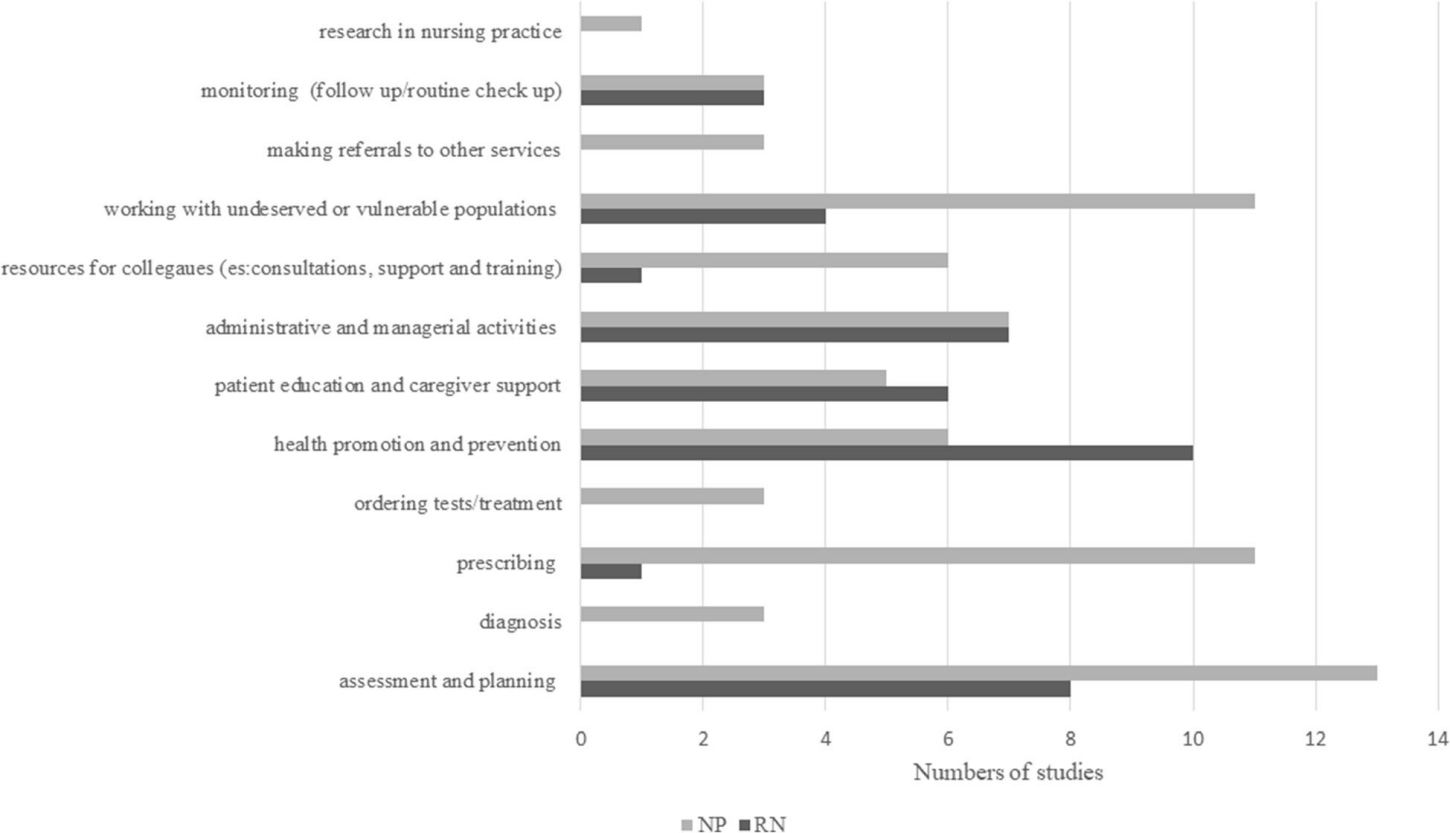
Stacked bar chart showing tasks reported for nurse practitioner and registered nurse. NP-nurse practitioner, RN-registered nurse

The main tasks carried out by nurse practitioners (NPs) and registered nurses (RNs) are illustrated in Fig. 2. All nurses worked in primary care settings, including general practice, health care centers, and rural/remote areas.

### Factors influencing implementation

The frequency of identification of barriers and facilitators in each domain is summarized in Table 3, while the specific determinants can be found in Additional file 3.

**Table 3:** Barriers and facilitators in each CFIR domain

Domain	Themes	Barriers	Facilitators
		N° of studies (%)	N° of studies (%)
1. Intervention Characteristic	scope of practice	16 (28,6)	13 (23,2)
	adaptability	0	1 (1,8)
	trialability	0	1 (1,8)
	workload	7 (12,5)	0
	education	14 (25)	7 (12,5)
	funding	11 (19,6)	2 (3,6)
2. Outer setting	patient factors	6 (10,7)	21 (37,5)
	external policies	5 (8,9)	0
3. Inner setting	culture	9 (16,1)	0
	workforce and organization	10 (17,9)	8 (14,3)
	communication	8 (14,3)	7 (12,5)
	implementation climate	26 (46,4)	28 (50)
	resources	9 (16,1)	1 (1,8)
4. Individual characteristics	team acceptance	30 (53,6)	24 (42,9)
	self confidence	4 (7,1)	3 (5,4)
	personal attributes	1 (1,8)	2 (3,6)
	individual stage of change	2 (3,6)	0
5. Process	planning	2 (3,6)	4 (7,1)
	stakeholder engagement	4 (7,1)	8 (14,3)
	development and implementation	2 (3,6)	8 (14,3)
	evaluation	4 (7,1)	2 (3,6)

The integrative review identified similar barriers and facilitators for both advanced role and a general nursing role. When factors are more referred to APN, we clearly indicated in the text. The main factors are listened below.

### Intervention characteristics

#### Barriers

With regard to the CFIR domain, nurses pointed to four main factors affecting nurses’ role implementation: 1) scope of practice; 2) nursing workload; 3) nursing education; and 4) funding.

*Restrictions of nurse scope of practice and autonomy* was the most frequently reported barrier to APN role implementation [21, 23, 24, 28, 31, 35, 44, 45, 47, 48, 53, 55, 56]. Arbitrary laws [31], state restriction, hospital regulations [28], and health care professionals’ expectations [35, 55] all contributed to restrict the independence of nurses and limit the full potential of their roles. For instance, some regulations required nurses to be supervised by physicians when exercising their prescriptive authority [38–40]. In addition, physicians often advocated the use of certain protocols [21] or required their supervision [45] through collaborative practice agreements [23, 31].

Other studies identified *excessive caseload numbers and complex cases* as barriers [25, 30, 32, 57, 58] to care provision [33, 71]. Furthermore, *patient care complexity*, alongside other non-clinical functions—mainly administrative and/or bureaucratic—, further increases the nurses’ workload [57].

*Education* was identified as a barrier to nurse’s role development in 13 studies. In particular, nurses expressed their concerns about the educational programs available to them, often questioning the adequacy of the training received [41, 56, 63], deemed insufficient to help them develop the skills required [25, 45, 62, 65, 70]. Nurses also complained about the existence of barriers to training opportunities and ongoing education [50], such as the lack of information regarding course availability [26], the difficulty in taking time off work to attend courses [26, 54], the need to travel long distance to reach the location where the course was being taught [32], and the lack of funding to cover education-related expenses [26, 51]. In regard to the latter, *funding to sustain the nurse position* was regarded as a barrier to nurse’s role implementation across 11 studies [21, 23, 36, 39, 42–44, 50, 52, 54, 66].

#### Facilitators

Nurses mainly indicated two facilitators of nurse’s role implementation: *i)* adaptability of the nursing role to the existing context [53] and *ii)* trialability [46]. Education and training were also reported as factors facilitating nurse’s role implementation. Educational resources such as master’s degree programs were generally thought to improve nurses’ clinical skills and provide job retraining opportunities, especially in primary care settings [26, 29, 36, 46]. Moreover, additional experiences, such as residency or fellowship programs after graduation, were felt as supporting role transitions in primary care [30]. One study reported that motivating nurses to study represented an additional important factor in attaining advanced practice levels [62]. Another facilitator was represented by nurses being satisfied with their full scope of practice [24–29] or working autonomously [27, 30–33]. Other facilitators included expanding nurse’s practice to carry out tasks normally performed by physicians [29, 35, 36] or putting nurses in charge of the communications between the patient and other care providers [29, 34].

### Outer setting

#### Barriers

*Patient-related factors* were reported as key barriers across several studies. From a patient perspective, one of the main factors negatively impacting the acceptance of the nursing role was the lack of knowledge and understanding of such role [42, 48, 56, 68, 69, 72]. Other factors included negative patients’ prior experience [68] and patients’ preference and medical condition [68, 69].

Five studies analyzing external policies from a nurse perspective identified prescribing restrictions [38–40] and remuneration policies [46, 48] as barriers to nurse’s role implementation.

#### Facilitators

Also in this case, most of the facilitators identified were related to *patient-related factors*. Generally, the care provided by nurses was regarded by patients as highly satisfactory [21, 41, 50, 65, 67] due to the many advantages it afforded, such as a more patient-centered communication [46, 50, 62, 68, 69] and the provision of personalized solutions to better meet their needs [25, 35, 36, 57]. Patients also described how their access to care would be quicker and easier [34, 50]. Several studies emphasized the patients’ acceptance of the nursing role [23, 36, 48, 63, 66, 68] thanks to knowledge and role recognition [59, 61] and nurse-community connection [50].

### Inner setting

#### Barriers

Barriers identified across studies were linked to organizational factors and were reported by different health care professionals (i.e., nurses, managers, and doctors).

*Recruitment and retention* of nurses were viewed as barriers due to the difficulty in recruiting and retaining qualified nurses [20, 29, 62, 65]. Organizational factors, such as lack of long-term human resource planning [52] and career opportunities [62] as well as uncertain employment [20, 26, 29, 55], all negatively influenced nursing role implementation. This barrier quite often led to high staff turnover among nurses [20] and increased intention to leave, especially among newly hired nurses [20, 72].

A few studies referred to the *organization’s culture, hierarchical structure* [29, 36, 48], and *difficulties in adopting a flexible approach to service delivery* [73] as main barriers to nurse’s role implementation. The nursing practice was overshadowed by the more dominant medical model [51, 58, 61, 63], prioritizing medical solutions to health problems rather than promoting patient wellness-centered care [35, 43].

The *nature and quality of communications* were among the environmental factors regarded as barriers to information access and support in rural areas. These were mainly due to isolation [32, 33], poor internet connection, and lack of electricity to run equipment [64]. Also, lack of information sharing between staff administrators and health professionals was associated with negative consequences [38, 64, 72]. Some studies reported that lack of shared understanding of the patients’ needs affected the team’s ability to provide care [57, 70, 71].

*Unfavorable implementation climate* was the most frequently reported barrier to nurse’s role implementation. The professional *relationship between health workers and other inter-professional workers* [22, 41, 42, 56] along with the lack of regulation of nursing role [22, 41, 42] hindered nurse’s role implementation [42]. In particular, the *lack of professional collaboration* was described as a strong obstacle to nurse’s role development [24, 29, 39, 41, 42, 48, 67, 74], with nurses emphasizing how counselors and secondary care providers would often refuse their referrals [24, 39, 41, 42, 48, 67, 74] or choose not to share with them critical information [41]. Among the causes of professional collaboration breakdown was the lack of support from physicians, managers, and administrative staff [26, 30, 33, 43, 44, 64, 72]. In general, nurses felt that they had not received enough collegial and managerial support [26], the same level of access to resources as that granted to physicians [38, 40], or the same respect as that paid to their peers [30, 72]. Consequently, nurses complained about the invisibility of their role in the community [22, 38, 72].

*Professional isolation* of nurses was reported as being an additional barrier in seven studies [24, 30, 32, 33, 50, 51, 64] due to the lack of integration with other health professionals in the workplace [32, 51]. These studies also pointed to the fact that the common goals were neither *shared with nor clearly communicated* to nurses by their employers [30, 32]. Furthermore, the contractual context was also shown to influence the climate as the lack of a reward and incentive system [20, 30] negatively affected the nurses’ morale [30, 55]. Lastly, according to several studies, the *lack of resources* was among the barriers to nurse’s role implementation [20, 29, 34, 36, 38, 39, 56, 57, 70].

#### Facilitators

Facilitators mainly referred to challenges for workforce development, nature and quality of communication, and implementation climate. Specifically, nurses reported that workforce challenges in primary care settings, such as changing patient case-mix [20, 42] and shortages of primary care providers [26, 50], favored nurse’s role development. Nurses also reported that communication strategies and technology helped them establish a relationship between primary and secondary care. On-call systems connecting healthcare professionals, telemedicine equipment, and team sharing of patient information, including case-reviews, were all crucial to the continuity of care [59, 64]. This is consistent with findings from other studies showing the importance of regular communication—preferably using the same electronic patient records—in the collaboration and coordination among health care professionals [34, 42, 50, 56].

Professional trust, mutual respect, and a close doctor-nurse relationship were also seen as facilitators of nurse’s role implementation and collaboration among nurses [31, 32, 42, 46, 50, 51, 56, 61]. In addition, inter-professional relationships and team working played a key role in facilitating nurse’s role development [25, 27, 35, 39, 41, 43, 48, 58]. This process was even more pronounced when nurses felt trusted and supported by physicians, pharmacists, managers, and colleagues [23, 24, 26, 29, 31, 38, 48, 64, 71]. Also mentoring, mainly from doctors and colleagues, was central to providing support during transition into the new role [26, 30, 39, 41, 44, 64].

### Characteristics of individuals

#### Barriers

Barriers identified across studies were primarily linked to poor team acceptance and low self-esteem among nurses. For instance, physicians’ resistance [23, 42, 56] was associated with lack of role clarity and concern about nursing practice [24–26, 30, 36, 38, 43–51, 66, 72]. Moreover, there was consensus among nurses, administrative staff, and team members that healthcare professionals were often not fully aware of the scope of the nursing practice [21, 28–30, 39, 45, 52, 53, 66]. In addition, physicians expressed lack of trust in nurses’ skills and knowledge [29, 36, 45, 47, 51, 54, 66, 72] and were concerned about their workload, nurse-doctor competition, and fragmentation and duplication of services [51, 52, 66], especially when the two roles were perceived as overlapping. The other major barrier was nurse self-doubt [44, 47]. In one study, nurses reported that they felt uncertain when colleagues did not regarded them as a resource [61].

#### Facilitators

Clarity and understanding of the nursing role were identified as crucial factors to gain the physicians’ acceptance [61]. The nursing role was more easily understood once doctors had previous nurse-doctor collaboration experiences [23, 26, 41, 52].

From a physician’s perspective, there were some motivations to employ nurses in primary care, including complementary relationships [52, 74] and enhanced quality and delivery of healthcare [28, 42, 66, 67]. Many physicians were satisfied with their collaboration with nurses [31, 34, 45, 50]. Consistently, other studies reported that nurse’s role in primary care settings reduced the physicians’ workload [21, 42, 46, 62], allowing these latter to focus on other more complex cases [42, 45]. Fittingly, nurses felt that they were instrumental in improving quality of care and increasing patient safety [31, 33, 35, 46, 48, 52, 59, 62] and considered their work to be valuable and worthy. Nurses expressed their satisfaction in providing more than patient care compared to other healthcare professionals [25, 41]. Finally, nurses were confident in their skills and knowledge [49] and aware of their own limits [31, 46].

### Process

#### Barriers

Process barriers were related to the lack of planning regarding nurse’s role utilization. In particular, it was unclear how care services would be adapted to meet changing needs [33, 73]. Furthermore, nurses often complained about the absence of clear leadership [71], top-down approach [56], and evaluation criteria. In two studies, nurses admitted their difficulties in identifying suitable tools to measure the outcome of their contributions [25, 59].

#### Facilitators

Few studies highlighted the importance of developing an implementation plan with a focus on workforce integration. Review of the existing nursing service, definition of roles and functions, and team involvement were useful considerations that guided planning [43, 56, 65]. Factors associated with better role development and integration were nurses’ involvement in developing their role (e.g., drafting job description) [24, 60], support from management, and strategic alliance with health authorities [24, 59, 61]. Universities were identified as external agents to the organization formally influencing role development [63]. The last facilitator was linked to the evaluation process. Nurses expressed the need to evaluate the effectiveness of their contribution [25] and identified research and audit mechanisms as resources to measure their professional outcome [41].

## Discussion

This integrative review includes 56 studies addressing barriers and facilitators during nurse’s role implementation in primary care settings. We have analyzed a large volume of information and experiences from the various stakeholders and identified several emerging factors influencing nurse’s role implementation strategies. Although we could not separate each contribution due to the miscellaneous participation in the studies, the different stakeholders’ perspectives allowed us to identify the specific barriers of and facilitators to nurse’s role implementation. These are summarized below.

### Barriers

Our synthesis shows that the major emerging themes regarding the barriers to nurse’s role implementation pertain to the following variables: *i)* the characteristics of the intervention; *ii)* the characteristics of the individuals; and *iii)* the inner setting of the healthcare professionals’ organization. Limiting factors were equally distributed among RNs and NPs, the two most represented nursing roles in primary care settings. Barriers related to the characteristics of the intervention are mainly due to the limited availability of and access to special education, which results in nurses lacking sufficient knowledge and skills to work in primary healthcare settings. Furthermore, key determinants of independent practice such as legislations and regulations also appear to influence nurse’s role implementation. Previous report showed that the restrictions to nurses’ full scope of practice mainly applied to prescribing for nurses in an advanced role [76], which forced them to collaborate with or be supervised by a physician. Moreover, our analysis indicates that nurse’s role implementation is dependent on the organizational setting in which it is embedded. Indeed, the decreased availability and retention of nurses are two phenomena predominantly seen in rural underserved areas, where lack of career opportunities and lower salaries compel nurses— especially newly hired ones—to relocate to other areas [77].

Consistent with previous findings [78], we show that lack of interprofessional collaboration and poor support from physicians and administrative staff has a negative impact on the implementation climate and healthcare provision, indicating that knowledge and beliefs of individuals belonging to an organization can influence individual acceptance of workforce change.

Overall, this review supports the notion that lack of role clarity among stakeholders is a significant and widespread barrier to optimal nurse’s role implementation [78]. This phenomenon is similar to what observed in the general practice where physicians protecting their professional boundaries and expertise can cause tension and confusion in the workplace [9].

### Facilitators

Major facilitators identified under the CFIR domains are linked to *i)* the characteristics of the intervention, *ii)* the inner setting of the organization, and *iii)* the implementation process. Key factors include prior planning for role introduction and nurses’ involvement in the early stage of role implementation. These findings further underscore the importance of the stakeholders’ involvement in driving the implementation process and building consensus on the nurse’s role [79]. More broadly, nurse’s role implementation should be preceded by in-depth reflections on the expected contribution of nurses to patient outcome achievement and team work [80].

With regard to challenges inherent in role development, job satisfaction and nurses’ access to high-quality education are the two main themes emerging among RNs and NPs. This is in line with a previous study showing that the standardization of nursing educational requirements—especially for nurses with advanced roles in the primary healthcare setting, such as NPs—supports role enactment [76]. Of note, the same study also highlights the importance of providing more interprofessional training while increasing the practice component of education.

Consistent with previous literature [78, 81], we find that building collaborative relationships in the workplace favors nurse’s role implementation and promotes nurses’ job satisfaction. Collaborative working does not always emerge spontaneously, which is in good agreement with Contandriopoulos et al. [80]. From a nurse’s perspective, respect, trust, and communication are the main pillars of successful doctor-nurse collaboration, as shown previously in the general practice [9]. Developing an effective collaboration between nurses and physicians may ultimately improve patient outcome thanks to the added value brought by nurses to the practice [82].

## Limitations

Even though this integrative review provides a comprehensive and accurate overview of the main facilitators of and barriers to nurse’s role implementation in the primary care setting. It is important to note that CFIR, used to selected constructs, identifies a list of factors within general domains that are believed to influence positively or negatively nurse’s role implementation, but does not rank factors in order of importance. Thus, we recommend to always consider multiple factors when implementing nurse’s role. In addition, although many aspects are transversal to the different countries involved in the study, the differences among contexts (e.g., political, social, cultural) and health systems make the results described herein non-standard. Another limitation is that the studies analyzed were published between 1996 and 2020. Thus, factors reported in studies published before or after this time period may not have been included. Lastly, as the factors contributing to nurse’s role implementation are quite complex, we may have missed some additional factors due to the language restrictions used in the inclusion criteria.

## Conclusions

From this integrative review, the following considerations emerge in a significant and transversal way: *i)* there is sub-optimal attention to the legislative and regulatory aspects governing the nursing profession; *ii)* there is only a partially complete regulation of the autonomy of the nursing profession; *iii)* there is paucity of studies on the role of professionals and various stakeholders in nurse’s role development and implementation in primary care; *iv)* there is lack of recognition of the nurse’s role and skills, especially within the multidisciplinary team; and *v)* there exist barriers to nurses’ training opportunities and ongoing education.

Overall, nurse’s role implementation appears to be a complex process influenced by numerous factors. Thus, there cannot be simple and linear recommendations to successfully develop and implement the nurse’s role in primary care. In this regard, the Medical Research Council framework [83, 84] has been used to guide the development of complex interventions, especially those related to nurse’s research and practice [85]. However, the fact that the facilitators may become barriers if not properly addressed poses some limitations to this approach. Indeed there is growing consensus on the need to consider—and simultaneously tackle—a number of factors influencing different domains (i.e., interprofessional, interpersonal, organizational, and systemic) when designing a tailored intervention. Likewise, our findings indicate that nurse’s role implementation needs to be contextualized, looking at barriers and facilitators and involving the inputs from different stakeholders as well as the legislative and regulatory aspects specific to the country of residence. It is only through this dynamic and context-dependent implementation process that nurses will be employed to strengthen the resilience of national healthcare systems around the world.

## Supplementary Information

**Additional file 1.** Search Strategy.

**Additional file 2.** Summary of key characteristics of the included studies.

**Additional file 3.** Facilitators and barriers identified by the studies mapped on to their corresponding CFIR domains and constructs.

## Data Availability

Data is available upon reasonable request. The detailed results of the assessment of all barriers and facilitators are available upon reasonable request. All requests relating to data should be addressed to erica.busca@uniupo.it

## Abbreviations

NCDs: Non-communicable diseases
WHO: World Health Organization
CFIR: Consolidating Framework for Research Implementation
APN: Advanced practice nurse
NP: Nurse practitioner, RN-registered nurse
