# Supplementary material for: Barriers and facilitators to the implementation of nurse’s role in primary care settings: an integrative review
Source: BMC Nurs. 2021 Sep 16;20:171. doi: 10.1186/s12912-021-00696-y (PMC8444166; doi:10.1186/s12912-021-00696-y)
Supplement: Supplementary file 1 — Additional file 1. Search Strategy. [file 12912_2021_696_MOESM1_ESM.docx]

**Additional File 1_Search Strategy**

**MEDLINE**

1. (((("Nurse Practitioners"[Mesh]) OR (((("nurse practitioner"[Title/Abstract]) OR "nurse practitioners"[Title/Abstract]) OR (("advanced nurse practitioner"[Title/Abstract] OR "advanced nurse practitioners"[Title/Abstract])))))) AND (((("Primary Health Care"[Mesh]) OR "primary health care"[Title/Abstract]) OR "Community Health Services"[Mesh]) OR "community health services"[Title/Abstract])) AND ((((((((implement*[Title/Abstract]) OR "introduction"[Title/Abstract]) OR improve*[Title/Abstract]) OR "experience"[Title/Abstract]) OR "barrier"[Title/Abstract]) OR "barriers"[Title/Abstract]) OR "facilitator"[Title/Abstract]) OR "facilitators"[Title/Abstract])
2. ((((((family health nurs*[Title/Abstract] OR (family nurs*[Title/Abstract] OR "Nurses, Community Health"[Mesh]) OR (community health nurs*[Title/Abstract] OR (district nurs*[Title/Abstract] OR "Nurses, Public Health"[Mesh]) OR (public health nurs*[Title/Abstract] OR "Family Nurse Practitioners"[Mesh]) OR (family nurse practitioner[Title/Abstract] OR family nurse practitioners[Title/Abstract])) OR (rural nurs*[Title/Abstract]))))))) AND (((((((((implement*[Title/Abstract]) OR introduction[Title/Abstract]) OR improve*[Title/Abstract]) OR "experience"[Title/Abstract]))) OR "barrier"[Title/Abstract]) OR "barriers"[Title/Abstract]) OR (("facilitator"[Title/Abstract] OR "facilitators"[Title/Abstract]))).
3. (((((((((("Primary Health Care"[Mesh]) OR "primary health care"[Title/Abstract]) OR "Community Health Services"[Mesh]) OR "community health services"[Title/Abstract])) AND ("Nurse's Role"[Mesh] OR "nurse's role"[Title/Abstract] OR "nurse's roles"[Title/Abstract] OR "nursing role"[Title/Abstract] OR "nursing roles"[Title/Abstract] OR "role nurses"[Title/Abstract] OR "role nursing"[Title/Abstract])))))) AND (((((((((implement*[Title/Abstract]) OR introduction[Title/Abstract]) OR improve*[Title/Abstract]) OR "experience"[Title/Abstract]))) OR "barrier"[Title/Abstract]) OR "barriers"[Title/Abstract]) OR (("facilitator"[Title/Abstract] OR "facilitators"[Title/Abstract])))

**CINAHL**

| **#** | **Query** |
| --- | --- |
| S23 | S9 AND S10 AND S22 |
| S22 | S20 OR S21 |
| S21 | TI nurs* role OR TI nurs* roles OR AB nurs* role OR AB nurs* roles |
| S20 | (MH "Nursing Role") |
| S19 | S10 AND S18 |
| S18 | S12 OR S13 OR S14 OR S15 OR S16 OR S17 |
| S17 | TI rural health nurs* OR TI rural nurs* OR AB rural health nurs* OR AB rural nurs* |
| S16 | (MH "Rural Health Nursing") |
| S15 | TI family nurse practitioner OR TI family nurse practitioners OR TI family health nurs* OR AB family nurse practitioner OR AB family nurse practitioners OR AB family health nurs* |
| S14 | (MH "Family Nurse Practitioners") |
| S13 | TI community health nurs* OR TI district nurs* OR TI public health nurs* OR AB community health nurs* OR AB district nurs* OR AB public health nurs* |
| S12 | (MH "Community Health Nursing+") |
| S11 | S8 AND S9 AND S10 |
| S10 | S6 OR S7 |
| S9 | S3 OR S4 OR S5 |
| S8 | S1 OR S2 |
| S7 | AB implement* OR AB introduction OR AB improve* OR AB experience OR AB barrier OR AB barriers OR AB facilitator OR AB facilitators |
| S6 | TI implement* OR TI introduction OR TI improve* OR TI experience OR TI barrier OR TI barriers OR TI facilitator OR TI facilitators |
| S5 | TI ( primary care or primary health care or primary healthcare ) OR TI community OR TI district OR TI community care OR TI community health care OR AB ( primary care or primary health care or primary healthcare ) OR AB community OR AB district OR AB community care OR AB community health care |
| S4 | (MH "Primary Health Care") |
| S3 | (MH "Community Health Services+") |
| S2 | TI nurse practitioner OR TI nurse practitioners OR AB nurse practitioner OR AB nurse practitioners |
| S1 | (MH "Nurse Practitioners+") OR (MH "Advanced Practice Nurses+") |
