# Supplementary material for: Barriers and facilitators to the implementation of nurse’s role in primary care settings: an integrative review
Source: BMC Nurs. 2021 Sep 16;20:171. doi: 10.1186/s12912-021-00696-y (PMC8444166; doi:10.1186/s12912-021-00696-y)
Supplement: Supplementary file 2 — Additional file 2. Summary of key characteristics of the included studies. [file 12912_2021_696_MOESM2_ESM.docx]

| Craswell, 2019 | Australia | MMS | To explore the reasons why some consumers express willingness to be seen by a NP when a medical practitioner is also available. | Adults (1,318) | PHC | 1.NPs  2.Master’s degree  3. First point of contact (refer patients to other health professionals, prescribe medications, diagnostic and blood tests) |
| --- | --- | --- | --- | --- | --- | --- |
| Drury, 2005 | Australia | QS | The purpose of this paper is to describe nursing roles of registered mental health nurses working in rural and remote areas. | CMHNs (5) | Community (rural and remote areas) | 1.CMHNs (RNs working in advance practice level)  2.Specific work experience in mental health.  3. Focus on community rather than just on the patient. CMHNs deliver holistic care. Responsibilities: therapeutic use of self, client assessment, ongoing monitoring, planning, implementing and evaluating care, crisis intervention, psychoeducation and caregiver support, liaison role. |
| Dunt, 1991 | Australia | CSS | To describe educational characteristics, career structure and work experience of community nurses. | Community nurses (695) | Community (practice area: community health centre, community-based district and visiting, maternal and child health, medical clinic, occupational health, ect.) | 1.Community nurses (nurse working outside hospital, nursing home or nursing education centre)(RNs)  3. Activities of a community nurse’s role: prevention and health promotion, assessment, direct patient care (maintenance of activities of daily living, provision of technical care and counselling), advocacy, administration (maintenance of records, liaison with other nurses or other health professionals), coordination and supervision, travel, case-sharing. |
| McCullough, 2020 | Australia | QS | This study described and explained from the perspective of nurses, the actions and interactions involved in the delivery of PHC in remote communities. | NPs (13), Remote Area Nurses (7), nursing academics (4) | PHC (remote areas) | 1.Remote Area Nurses (RN)  3.Care for the community and individuals, with a focus on illness prevention, equality of care. Role as a co-ordinator of care to facilitate access to health services. |
| Parker, 2013 | Australia | QS | To examine Australian health care consumers’ perceptions of nurse practitioners working in primary health care | 77 participants to focus groups | PHC | 1.NPs  2. RNs completed a masters’ degree  3.NPs performed an advanced and extended clinical role (assessment and management of clients, prescribing medication, ordering diagnostic intervention, and making direct referral). NPs have whole person approach to delivering health care. |

| Burgess, 2010  Burgess, 2011 | Canada | QS | We used a PAR approach to explore, from the perspectives of NPs the relevance of collaboration in advancing NP role integration in primary healthcare (PHC)  (focus on the effects of collaboration on NP role integration) | NPs (17) | PHC | 1.NPs  3. Collaborative relations with clients, colleagues and healthcare leaders. NPs developed their role as multi-faced and carry out complex client and underserved/marginalised groups and community assessment, apply evidence-based guidelines, prescribe and provide treatment, initiate health promotion and prevention programs,patient advocacy, link clients to various community resources. Holistic approach to care. |
| --- | --- | --- | --- | --- | --- | --- |

| Côté, 2019 | Canada | QS | To better understand the factors that impede or facilitate stakeholders in achieving an optimal use of the PHCNP role. | PHCNPs (27) | PHC | 1.PHCNPs (NPs) |
| --- | --- | --- | --- | --- | --- | --- |
| De Guzman, 2010 | Canada | CSS | Identify the NPs’ perceptions of barriers and facilitators associated with the implementation of their role in Ontario’s PHUs, the NPs’ job satisfaction, and the relationship between NP job satisfaction and practice dimensions. | NPs (28) | PHU (71.4% of NPs were practicing in sexual health programs. 21.4% were practicing in the prenatal/ postnatal care, 7.1% practiced in other areas, such as primary health care) | 1. PHCs (NPs)  2.BScN degree and post-baccalaureate (Primary Health Care) NP certificate.  3.Provide clinical care (69.4%), clerical work (7%) and education (7%). Most respondents (89.3%) worked in an area designated as being under-serviced for physicians. |
| Domm, 2019 | Canada | QS | To gain understanding about PHN perceptions of their evolving work and how PHN work was managed. | PHNs (42) | Urban and rural setting | 1.PHNs  3. Provide health assessment, health promotion and education, administer vaccines, linking role between patient and social support. |
| Gould, 2008 | Canada | QS | To investigate the experiences of nurse practitioners (NPs) 1 year after they were first introduced to a mostly rural Canadian province. | NPs (7) | Primary care (rural area) | 1. NPs  2.Majority of NPs were licensed within the past 2 years.Interviewees had worked as RNs for an average of 21.1 years (SD = 11.9, range = 3–40 years) before becoming NPs.  3. NPs spend time with patients, patient involvement, educating patients, as part of illness prevention goals but also in order to ensure adherence to prescribed regimens. History taking.. Team-working and holistic approach to patient care (housing, mental health problems, financial problems). |
| Hunter, 2016 | Canada | MMS | To determine benefits and challenges of a rural primary care NP role | Survey: 41 patients, 1 HCP, 4 physicians; Interviews: 14 patients, 1 HCP, 2 LLs and the NP. | Primary care (rural area) | 1.NPs  3. NPs provide follow-up for chronic patients, acute concern, routine check-up/prevention and collaborate with clinic colleagues and HCP. |
| Jean, 2019 | Canada  Spain | QS | To develop a comprehensive understanding of the contextual factors that influence the development and implementation of APN in two countries, Canada and Spain | Nurses (32), medical doctors (10), Psychologists (2), allied health professionals (2), other (1)* | Community | 1.APNs  2.The majority had a master’s degree (34%) or a PhD (25.5%). |
| Martin-Misener,  2010 | Canada | MMS | To describe how rural health board chairpersons and health-care providers define the role of NPs in Nova Scotia. It summarizes their perspectives of the health needs of rural communities, the gaps in the current model of PHC services, the envisaged activities of NPs, and the facilitators of and barriers to NP role implementation. | Chairpersons (51) | PHC (Rural Area) | 1. Rural NPs  3.NP was described as generalist, partial overlap with FP. Care for patients with common urgent health issues. Provide health promotion, prevention and chronic disease management, outreach, address physical, social and mental health concerns and counsel and educate patients. Community activities: liaison role (community and FPs and community services). Work in collaboration with FPs and other health-care providers (some settings requirement for formal collaborative agreement).  Most respondents(39%) indicated that NPs provided assessment and diagnosis activities . In addition, respondents reported that NP prescribed some medications (e.g. contraceptives, antibiotics), and performed procedures ( <30% of respondents). Fewer than 35% indicated that NPs carried out consultations and referral activities. 30% of respondents indicated that NPs performed community activities (mostly carried out by PHNs/ FPNs). |
| Reutter, 1996 | Canada | QS | To explore the public health nurse's perception of their business and experience | PHNs (28) | Community (different settings: well-child clinics, home visits, schools, rural areas) | 1.PHNs  2. 24 PHNs had baccalaureate degrees, 2 PHNs had a diploma in public health nursing, 2 PHNs had an RN diploma.  3.Family and community focus; health promotion (e.g. immunization, pre-natal and post-natal education and illness/injury prevention programme. |
| Sullivan-Bentz, 2010 | Canada | QS | To examine the influence of inter-professional relationships, particularly those with family physicians; explore the factors influencing and hindering successful transition into NP practice; and recommend ways to support new NP graduates | Anglophone & francophone NPs (23) & co-participants (physicians, NP colleagues, or administrators) (21) | PHC (rural and urban) | 1.PHCNPs (NPs)  2.Anglophone NPs: "All had bachelor’s degrees in nursing and 3 had master’s degrees." Francophone NPs: "One was master’s-prepared" |

| Burke, 2010 | Ireland | QS | This study investigated the experiences of community nurses working in four pilot teams. | PHNs (14), CRGNs (4), PNs (3), CMHN (1) | Primary Care | 1.PHNs  3.Collaborative relationships with other members of the primary care team. PHNs have geographical boundaries. PHNs treat an entire family, manage complex cases and provide health promotion. |
| --- | --- | --- | --- | --- | --- | --- |

| Zug, 2016 | Latin America and the Caribbean | CSS | To identify the current state of APN regulation, education, and practice in Latin America and the Caribbean and the perception of the APN role in PHC | Nursing leaders or key informants in their country (University employees, Educations, Ministry of Health employees, policymakers) (173) | PHC | 1.APNs  2.master’s degree is recommended” (The International Council of Nurses”. |
| --- | --- | --- | --- | --- | --- | --- |

| Adams, 2019 | New Zealand | QS | This paper reports on the barriers and facilitators to becoming a NP in rural PHC | NPs (11), NPs candidate (4) | PHC (rural): general practices, PHOs, DHB | 1.NPs  2.Clinical Master’s degree  3.Primary response in medical emergencies. Managing patients with long term conditions. Working collaboratively with GPs. |
| --- | --- | --- | --- | --- | --- | --- |
| Carryer, 2011 | New Zealand | QS | To explore the transition from rural nurse to NP. | Nurses (21) | Primary Care (rural) | 1.NPs  2.1 ‘authorised NP’, 1 unsuccessful application, 1 application pending. Master’s Degree -11 completed (not yet submitted an application), 2 commenced but not completed, 5 not started.  3.Broad scope of rural practice- across the lifespan. Community integration. |
| Carryer, 2017 | New Zealand | QS | To consider the alignment of the NP role in New Zaeland with the goals and aspirations of the many countries facing challenges to maintaining health service delivery and reducing health disparities. | NPs (13) | PHC (rural locations) | 1.NPs  2.Masters educated  3.Patient-centred approach attending to family, community, and social, political and economic factors, education, order laboratory tests, diagnose, prescribe and other tools, making the system work for patients.Collaborative model of care. |
| Mackay, 2003 | New Zaeland | MMS | To explore perceptions of GPs in the NDHB regarding the NP role, identifying their knowledge of and perceived problems with that role, and their experience of nurses in advanced practice. | GPs (47) | PHC | 1.NPs  2.NPs are educated through a clinically focused master’s degree programme and must meet the competencies set out by the nursing council.  3.Advance the scope of their nursing practice, expert practice, working collaboratively with other disciplines as well as across settings, leadership and consultancy in nursing, development and influence policy and nursing practice, research on nursing practice. Nurse prescribers (optional) (Nursing Council of New Zealand, 2001). GPs rated - health teaching to promote health or to prevent illness, home visits to do follow-up, evaluation of care, taking histories (favourable). Prescribing, ordering tests, physical examinations (least favourable). |

| Clancy, 2009 | Norway | QS | Describe and analyze local decision-makers’ views on public health nursing and to reflect on and discuss the relevance of those views to the future of public health nursing. | Politicians (5), administrators (6) | Community (municipality) | 1. PHNs  3. PHNs discover and refer problems and support and advise parents, school children and young people.  Collaboration with other professions (interprofessional and intraprofessional collaboration). Leader’s role. |
| --- | --- | --- | --- | --- | --- | --- |
| Lindblad, 2010 | Sweden | QS | To describe the first Swedish APNs’ and their supervising general practitioners’ (GPs) experiences of an APN’s role and scope of practice. | APNs (4), GPs who had supervised the APNs (5) | Primary Health Care Centres | 1.APNs  2.Advanced Clinical Nurse Specialist’s degree  3.Independent role, patient care acute health problems, such as infections (upper pulmonary infections, UTIs, otitis, dermatitis and skin problems).Right to prescribe medication and order treatment. |
| Ljungbeck, 2017 | Sweden | QS | To investigate the opinions of managers, doctors  and nurses in primary care and municipal healthcare about the role of ANPs in municipal healthcare. | Doctors in primary care(4) Managers(4)and nurses working in municipal healthcare(4) | Primary Care and Municipal healthcare | 1.Specialist nurse  2. The specialist nurses must have worked in municipal healthcare as a specialist nurse for at least two years to have gained the experience and understanding that advanced nursing care requires.  3. Nurses believed the ANP would increase patient safety as the ANP be clinically competent, provide leadership and collaborative practice. Continuity of care - follow the frail elderly through different types of services and take more responsibility for the patients regarding both nursing and medical care. Personalized and person-centred care. Managers believed ANPs could educate and support other nurses. Enable healthcare of the frail elderly. |
| Boman, 2019 | Sweden, Norway, Denmark | QS | To explore the feasibility of introducing GNSs in PHC | Older person (5), Nurses (5), Nurse leaders (5), Physician (5), Politicians (5) | PHC | 1.GNSs (NPs)  2. Master’s degree in advanced geriatric nursing  3. Care needs of the comorbid older patients. Focus on assessment (medical conditions, social and psychological factors, laboratory tests). Linking role |

| Gysin, 2019 | Switzerland | QS | To explore APNs and GPs views on introducing the APN role to Swiss primary care | APNs (9), GPs (4) | Primary care | 1.APNs  3.Focus on patients and their daily life, advanced care planning, technical patient care, coordination with the social sector. |
| --- | --- | --- | --- | --- | --- | --- |

| Parfitt,  2007 | Tajikistan | QS | To evaluate the progress of the implementation of Family Health Nursing as part of Tajikistan’s health service reforms. | FHNs interviewed (18), families, FPs (9). | PHC (five rural sites). | 1. FHNs.  2. Graduate certificate in Family Health Nurse (4 years curriculum)  3.One year after implementation. Implementation of FHNs was very variable across the five sites: some FNHs reported that there wasn’t any real change to their role or responsibilities, others referred that they spent more time on community activities and worked in more independent ways.  Five year after implementation. FHNs primary task was carried out a risk assessment for local families and drew up a plan of primary intervention (e.g. health promotion, delivering treatment or making direct referrals), in consultation or independently with the FP. FHNs were being seen as the community’s first point of contact with the health system. |
| --- | --- | --- | --- | --- | --- | --- |

| Lovink, 2018 | The Netherlands | QS | To describe how skill mix change is organised in daily practice, what influences it and what the effects are of introducing NPs, PAs or RNs into primary healthcare for older people. | GPs (9), NPs (10), PAs (5), RNs (10). | PHC (including general practice care and community care) | 1. NPs, PAs and RNs (district nurses)  2. NPs and PAs had EQF 7, RNs had EQF level 4,5 or 6.  3. NPs performed general consultations and medical care for patients from all ages (medical anamnesis, physical examination, prescription of medication, psychosocial support, referral to other discipline). RNs provided nursing care to patients with chronic diseases (nursing anamnesis,nursing procedures, psychosocial support.health education and monitoring)  NPs and RNs performed proactive healthcare for older people (preventive home visit, screening on frailty, organisation of multidisciplinary meetings). |
| --- | --- | --- | --- | --- | --- | --- |
| Van der Biezen, 2017 | The Netherlands | QS | To provide insight into factors influencing the decision of GPs and managers to train and employ a PA/NP within their organisation. | GPs (32), managers (7) | Primary Care | 1.NPs  2.Master’s programme  3. GPs expressed different views as to whether NPs should treat chronic patients, acute problems, palliative care, gynaecologic complaints and care for elderly or young children. NPs treat minor ailments (e.g. dermatology, ear nose and throat complaints) provide social home visits and postoperative consultations. Focus on direct patient care first. Indirect tasks ( meetings with other primary care professionals, coordination of elderly care, developing protocols and training support staff) were likely to be considered when NP would be more experienced. |

| Carr, 2002 | England (UK) | CSS | To investigate GPs perceptions of the NP role in one NHS region. | GPs (225) | Primary Care | 1.NPs  3.Activities that potentially should be carried out with/without protocols: diagnosis of disease in adults, health promotion, triage, prescribing, treatment, minor illness and chronic disease management. Reservations about the treatment of children. |
| --- | --- | --- | --- | --- | --- | --- |
| Carr 2005 | England (UK) | QS | To explore the development of public health nursing in a PCT that focused around the evaluation of a newly introduced PHN role. | HVs/PHNs and stakeholder (e.g. medical consultant, social worker, PCT Director of nursing) | Primary Care (PCT) | 1.PHNs  3. Public health activity. |
| Crawford, 2001 | England (UK) | CSS | To explore the impact of placing CMHNs full-time within primary care practices. | Primary care personnel (including reception and administration staff, GPs, nurses and health visitors)(38) | Primary care practices | 1. CMHNs  3.Role perceived: liaising with the PHC team, counselling and general support. CMHNs offer clinical assessment and care, crisis intervention, psychological interventions and support, medication management and follow up. |
| Drennan, 2011 | England (UK) | MMS | To examine the factors affecting the extent to which English policy on the introduction of CMs for people with chronic conditions was implemented. | Directors of Nursing (41), stakeholders (e.g. managers of CMs, patient rapresentatives, GPs) (30) | Primary Care (PCTs) | 1.CMs  3.nurse case manager role to support people with multiple long-term conditions: assessment of physical, mental and social care needs; review medication and prescribe medicines; clinical care and health-promoting interventions; coordination; patient and caregiver education. |
| Drennan, 2019 | England (UK) | QS | This study investigated the factors influencing workforce development of the district nurse service in metropolitan areas. | Senior nurses in provider organizations (6), CCG senior nurses (8) | Metropolitan areas | 1.RNs  3. nurses provided services to housebound adults. |
| Kipping, 1998 | England (UK) | MMS | To explore MHNs expectations and experiences of working in the community. | MHNs (survey: 447; interviews: 12) | Community (community mental health centre, residential homes, group homes and hostels and crisi teams | 1. MHNs |
| MacDonald, 2005 | England (UK) | QS | To establish whether or not community nurse practitioners were able to achieve a 'higher level of practice', as articulated by the United Kingdom Central Council | Community NPs (22) & Clinical Managers (GPs, Senior Community Nurse, Community Nurse Managers, Community Managers) (17) | Community | 1.Community NPs  2.Most participants had completed the RCN Nurse Practitioner diploma, or the BSc Honours Health Studies(Primary Health Care) RCN degree. Four participants were completing the franchised RCN degree course.  3.Working in GP practices, homeless services, minor treatment centres. NPs who achieved a 'higher level of practice"/ 'intermediate stage' held consultations with patients with 'undifferentiated diagnoses' and a wide variety of acute, minor and chronic illnesses. NPs worked with 'GP overlap' were the first points of contact and were able to undertake physical examination. NPs working with 'restricted practice'; attending to patients who had had their illness previously diagnosed by the GP (minor illness:women's health problems and chronic disease such as asthma and diabetes). Informal teaching role. |
| Main, 2007 | England (UK) | QS | To explore how health professionals perceive the current and potential role of nurse practitioners in primary care. | Organisations with different models of PC delivery (5): NPs, GPs, PNs & managers (21) | Primary Care (PCT) | 1.NPs  2.ANP is ‘a RN who has undertaken a specific course of study of at least first-degree (honours) level.’MSc (n=1), BSc (n=1), diploma (n=2), completing a qualification (n=4).  3.Prescribing is not part of the NP qualification. |
| Perry, 2005 | England (UK) | QS | To explore the role of a nurse practitioner in primary care, particularly whether the provision of a nurse practitioner facilitated access to care that met the needs of patients. | PHCT (10) (nursing staff, GPs, practice manager, reception staff); patients (14) | Primary care | 1. NPs  2.NP had undertaken an accredited nurse practitioner master's degree  3. Increased access to services,in terms of meeting patients needs (e.g. address social and economic needs), and number of appointments, continuing throughout the day. |
| Plews, 2000 | England (UK) | QS | To examine the understanding and practice of public health nursing throughout the Region and identify both the constraints and opportunities that might help develop public health nursing. | Health Authorities (10), Acute Trusts (18), Community Trusts(18)** | Community | 1.PHNs (HV and School nurses)  3.PHNs worked in alliance with other agencies to develop a collaborative approach to health based on needs assessment and health promotion activities. |
| Price, 2003 | England (UK) | QS | To explore other NPs’ roles in referral with the purpose of clarifying the issues and stimulating debate. | Consultants in the local general hospital (6), PCNPs (7); GP registrars (10); lecturers in a NP programm (2) | Primary care and secondary care | 1.NPs  2. RCN Nurse Practitioner Diploma, or a Bachelors Degree in Nursing (NP pathway)  3. The gatekeeper role. Collaborative relationship with a GP. |
| Rapport, 1997 | Britain (UK) | QS | To explore the responses of primary health care professionals and their patients to changes taking place within the community. | Participants (43): district nursing team leaders and members, district nursing officers, GPs, a fundholding practice manager, social workers, a social work manager, the chairmen and directors of private nursing homes, and patients. | Community | 1. DNs  3. DNs are involved in a variety of direct and indirect patient care activities (patient referral and assessment, continuing of care and assessment for aids and equipment). Collaboration with other professionals. |
| Wilson, 2002 | England (UK) | QS | To explore the views of British GPs regarding their attitudes toward developing an APN role in general practice. | GPs (25) | Primary care (medical practices) | 1.NPs |

| Athey, 2016 | USA | CSS | To examine factors that predict NP job satisfaction | NPs (8,311) | Primary Care (ambulatory clinics,hospital and other settings) | 1. NPs  2.Less than master’s (5.9%), Master’s degree (89.5%), Doctorate (4.6%)  3.Collaborative relationships with physicians. Majority of NPs reported that their skills were being fully utilized. NPs who worked in ambulatory care settings reported more autonomy than those in hospitals. |
| --- | --- | --- | --- | --- | --- | --- |
| Conger, 2008 | USA | QS | To examine rural connectedness versus disconnectedness. | Nursing master’s graduate (RHS, clinical specialist with a rural focus and FNPs)(30) | Primary Care (rural areas) | 1.APNs  2.Masters degree. |
| Donelan, 2013 | USA | CSS | To survey the role of nurse practitioners in PC and the likely effects on the health care system of expanding the supply of NP and the scope of their practice. | NPs (467), Primary care physicians (505) | Primary Care | 1.PCNPs (NPs)  2.Licensed clinicians who had been trained in a primary care specialty, were actively working in primary care practice, and were providing direct patient care.  3.74.9% NPs believed they were currently able to practice “to the full extent of their education and training.Collaborative practice. Provide most primary care services with physicians. 28.3% physicians agreed NPs provide care for complex patients (multi-morbidity/ not well controlled). |
| Faraz, 2017  Faraz, 2019 | USA | CSS | To identify factors associated with a successful transition and turnover intention of novice NPs in the PC workforce. | NPs(177) | Primary care | 1.NPs  2.141 NPs held a master’s degree in nursing. NP program type: Traditional master (102); Accelerated master (32); BSN master (29) DNP (9) Other (7) |
| Fletcher, 2007  Fletcher, 2011 | USA | CSS | To compare the quality of care provided by NPs and physicians (MDs) for patients with hypertension and/ or diabetes within the VA health care system; and to assess differences in perceptions, if any, between NPs and physicians regarding the role and scope of practice of NPs within the VA health care system. | NPs (74), Physicians (79) | Primary Care | 1.NPs  3. NPs reported working in a variety of roles with varying responsibility and independence (clinical, administrative, managing chronic/ acute patients, conducting assessments, planning care, adding/ changing medications. Assisting/ collaborating with physicians.). Activities (reported by physicians): patient education, take history, medication review and evaluation, care for simple cases, administrative/managerial role. |
| Hansen-Turton, 2013 | USA | CSS | Report the results of a recent assessment of the credentialing and reimbursement practices of the largest MCOs in the United States. | Representatives from HMOs (258) | Primary Care | 1.NPs |
| Jarrell, 2016 | USA | CSS | To assess the professional development and mentorship needs of the NPs as a first step toward development of effective programs to meet needs. | NPs (198):113 are nurses practicing in primary care | Primary care | 1. NPs  2. 33 had a doctorate of nursing practice or other doctoral degree, and 165 had master’s degrees |
| Kraus, 2017 | USA | QS | To provide a rich descriptive understanding of how doctors and NPs feel about NP practice in primary care, particularly their independent practice, and why. | Physicians (15), NPs (15) | Primary care (private and academic practices) | 1. NPs  2. PhDs (n=2), DNPs (n=2),enrolled in DNP programs (n=2); all others held a master’s degree or graduate-level certificate. |
| Poghosyan, 2013 | USA | QS | To describe NP roles and responsibilities as PCPs in Massachusetts and their perceptions about the barriers and facilitators of their SOP. | PCNPs (23) | Primary Care (community health centre, private physician practices, ambulatory, and hospital-affiliated outpatient clinics). | 1.NPs (PCNPs)  2.Masters degree  3.NPs provide comprehensive primary care (e.g. preventative, episodic and chronic care).NPs have an holistic approach to patients (e.g. family dynamics).Some NPs have specialized and see patients with specific conditions. NPs have prescriptive authority. |
| Poghosyan, 2017 | USA | CSS | Examine and compare the NP patient panel, job satisfaction, turnover intentions, and organizational structures of NPs with less than three (newly hired) and more than three (experienced) years of experience in their current job. | NPs (278) | Primary Care  (urban rural and suburban)  (community health centre, private physician office, and hospital-affiliated outpatient clinics) | 1.NPs (newly hired and experienced)  2. Master’s degree/post-master’s certificate (203) and Doctor of Nursing Practice (18) |
| Weiland, 2015 | USA | QS | To elicit an understanding of the meaning of autonomy as interpreted by NPs through the lived experiences of everyday practice | NPs (9) | Primary care | 1. NPs  2.Master’s NP (8); DNP (1). Advanced practice certification: Family NP (7); Adult NP (1); Pediatric NP (1)  3.Autonomy for patient care (restricted in Oklahoma – require physician supervision for prescription) |

* We only used data from Canada, because the practice setting of APN development and implementation was the community. In contrast, a majority of participants from Spain were employed in hospital settings.

** We only used data from nurses worked in Community Trust.

Legend: APN-Advanced Nurse Practitioner; BSN- Bachelor of Science in Nursing; CCG- Clinical Commissioning Group; CM-Community Matron; CMHN-Community Mental Health Nurse; CRGN-Community Registered General Nurse; CSS-Cross Sectional Study; DN-District Nurse; DNP-Doctorate in Nursing Programme/ Doctor in Nursing Practice; DHB-District Health Board; FHN-Family Health Nurse; FNP-Family Nurse Practitioner; FP-Family Physician; GNS-Geriatric Nurse Practitioner; GP-General Practitioner; HCP-Health Care Professional; HPN-Health Plan Nurse; LL-Local town/administration/healthcare leader; MCO-Managed Care Organization; MHN-Mental Health Nurse; MMS-Mixed Methods Study; NDHB-Northland District Health Board; NHS-National Health Service; NP-Nurse Practitioner; PA-Physician Assistants; PCNP-Primary Care Nurse Practitioner; PCT-Primary Care Trust; PCP-Primary Care Provider; PHC-Primary Health Care; PHCT-Primary Healthcare Team; PHCNP- Primary Healthcare Nurse Practitioner; PHN-Public Health Nurse; PHO-Primary Health Organization; PHU-Public Health Unit; PN-Practice Nurse; QD-Quantitative design; QS-Qualitative Study; RHN-Rural Health Specialists; RN-Registered Nurse; SOP-Scope of Practice; VA-Veterans Affairs
