# Supplementary material for: Barriers and facilitators to the implementation of nurse’s role in primary care settings: an integrative review
Source: BMC Nurs. 2021 Sep 16;20:171. doi: 10.1186/s12912-021-00696-y (PMC8444166; doi:10.1186/s12912-021-00696-y)
Supplement: Supplementary file 3 — Additional file 3. Facilitators and barriers identified by the studies mapped on to their corresponding CFIR domains and constructs. [file 12912_2021_696_MOESM3_ESM.docx]

**Additional File 3_Facilitators and barriers identified by the studies mapped on to their corresponding CFIR domains and constructs**

| **Construct** | **Domain** | **Facilitator [reference number]** | **Barrier [reference number]** |
| --- | --- | --- | --- |
| **1. Intervention**  **Characteristic** | | | |
|  | A. Intervention source |  |  |
|  | B. Evidence strength &  Quality |  |  |
|  | C. Relative advantage | “Nurse full scope of practice”(1–6) “professional autonomy also related to work settings”(2,7–10) “linking role between patients and health care professionals” (3,11)“task-shifting”(3,12,13) “NPs credentialed as primary care providers”(14) | “restrictions of nurse scope of practice”(4,5,21–25,8,12,15–20) “formal collaborative practice agreement”“physician supervision, when the doctor-nurse relationship was legislated ”(16,26,27) |
|  | D. Adapability | “adapting the nurse’s role to existing context”(25) |  |
|  | E. Trialability | “trying out the new model in small-scale projects”(29) |  |
|  | F. Complexity |  | "calculating staffing ratios"(30)”caseload numbers and composition”(6,7,9,31) “nurses don’t have enough time for patients’ visits or other tasks part of their role due to the heavy caseloads”(7,10,32)“the increase in administrative duties”(31) |
|  | G. Design quality &  packaging | “completion of previous RN portfolio as a part of professional recognition”(1) “job description”(23)  *education and training*  “motivate nurses to study”(33)“master’s degree program in advanced nursing”(1,3,29) “re-training program to equip already-qualified nurses”(13) “residency or fellowship program after graduation”(7) “maintaining specifical technical skills”(34) | “nurses lack of competence and skills”(6,19,33,35,36)”  *education and training*  “academic education not adapted to clinical need”(23) “the preparedness level of the faculties in teaching an advanced level of PHC or community health”(37) “lack of ongoing education specific to NPs needs”(38)  *obstacle to training*  “inability to attend training/ ongoing education (39) due to the distance (rural nurses)(9) the heavy caseloads(17) the lack of funding(1,34,40)”“Inconsistent information available for the planning and completion of educational programme (Master), and difficulties negotiating time”(1) |
|  | H. Cost | “contractual agreement with physicians in order to work around reimbursement barriers”(16)“independent APN tariff, official legitimization to use the TARMED”(29) | “GPs funding mechanism”(15,17,27,41,42)“GPs lack of financial remuneration provided for training and employing NPs” (21)  “under-resourced health system: decline in funds for public services”(13) “Poor availability of funds to cover NP services and positions”(24,39,43) “financial uncertainties”(44)  “no clear regulation provided for billing for nurses services”(29) |
| **2. Outer setting** |  |  |  |
|  | A. Patient needs &  Resources | “Patients satisfaction”(21,36,38,39,45)  *benefits and advantages for patients*  “nurses were able to meet the needs of patients because they see patients in their environment(6,13,31), they know client’s networks(9),they are accessible through telephone dedicated line and flexible in scheduling appointments(6) and service delivery(11,39), they are good listeners and trustworthy(46) and they devise a service that is contextual (solution and proposal was adapted to meet the needs and fit the resources within the local community)” (12)“nurse have time for the patient”(29,33,39,46,47) “NP asked more questions and explained things in more detail than GPs ”(45)  *acceptability*  “patient acceptance for nurses providing PHC(13,22,24,37,43,47) “nurse role recognition (48)“nurse connection to the rural community”(39) “NP-client interactions and role clarity are an important step in gaining acceptance”(49) | *acceptability*  “patients lack of knowledge and understanding of the NP role (22,23,42,47,50)” “Confusion around understandings of how a NP differed from others type of nurses and GP”(22,46) “lack of clarity regarding reasons and circumstances to consult a NP rather than a GP”(46) “patients lack of willingness to be seen by a NP for conditions they perceive serious or complex” (46,47) “patients prior bad experience”(47) “patients opposition”(23)“appropriate training was a major concern in terms of acceptability of the nurse role”(46) |
|  | B. Cosmopolitanism |  |  |
|  | C. Peer pressure |  |  |
|  | D. External policies &  incentives |  | “lack of formal/status recognition of the nurse role in advanced level practice”(9)“political uncertainties (regarding legislation and scope of practice)”(44)“lack of a professional register (non-recognition of the NP role by the NMC)”(40) “health care reforms”(18)“fee-for-services system”(22) |
| **3. Inner setting** |  |  |  |
|  | A. Structural characteristics | “nurses and doctors were a part of the same organization”(33)  “nurses and other health professionals working in the same environment”(51)  *challenges for workforce development (nursing services)*  “changing patient case-mix”(34,42) “shortage of primary care providers”(1,39) “patient demand for PHC services”(37)  “new job opportunities and opportunities for career development”(10) | “lack of long-term organisation and workforce planning” (44) short-term service contracts”(34) “uncertainty about professional future”(18) “uncertainty about employment opportunities as an NP”(1,3)  “recruiting suitably qualified nurses(3,33,36) and retaining them(34)”  “high staff turnover and high use of agency staff”(34) “intention to leave”(34,50) “any career opportunities”(33) “lack of remuneration of nurse’s overtime hours”(18,23) |
|  | B. Networks & Communications | “nurses connection on national level facilitated sharing nursing practices”(32) “journal club with other health professionals”(52)  “public awareness campaign was mentioned as a strategy for increasing public recognition”(48)  “regular and effective communication(11,23,39,42) preferably use the same (electronic) patient records(42)”  “support networks to get patient discharge information from the regional hospital”(52) “Communication strategies and equipment: one call’system to connect the rural provider with a specialist, e-mail, telemedicine equipment”(52) “informal team-sharing of client information and formal case-reviews”(48) | “environmental factors: poor internet connection, isolation, lack of electricity to run equipment” (52) “limited access to immediate collegial support (geographical isolation and lack of available services)”(9,10)  “lack or poor relations with administration and physician”(26,50,52) “lack of shared understanding of the conditions and patients’ needs that impact on the ability to provide care”(31,32,35) |
|  | C. Culture |  | “hierarchical structure”(3,13,22) “practice focused on individual basis” (28) “biomedical/treatment model”(30,37,40,49)  “giving primacy to medical solutions rather than person centred care”(12)“Health Authorities worked on a medical model of health care”(41) |
|  | D. Implementation climate | *interprofessional relationship and collaboration*  ”interdisciplinary collaboration”(2,6,12,22,30,42)“development of interprofessional teams”(12,27,30,38,41) “intersectoral collaboration and working”(41) “collaborative working(8,9,23,52) and interaction/relations(39)”  “support from GPs (8,22,24,26), pharmacists and specialists in a particular area (52), managers and Directors of Nursing”(1,3,4) and colleagues”(32)”availability of mentoring (colleague or GP)”(1,7,15,27,38,42,52)  “trusting relationship with physicians and others”(4,16,23,29,42,49)  *organizational incentives*  “NPs were reimbursement at the rate of PCPs (HMO)”(14)  *goal and feedback*  “leadership and vision of how the care model should be developed”(23)  *compatibility*  “clarify what kind of tasks a nurse should have compared with other roles”(33) “clear role and responsibility distinction for current nursing service”(37) | *interprofessional relationship and collaboration*  “interprofessional competition”(23,42) “lack of regulations of the NP role” (38) “lack of clear boundaries for the NPs’ and RNs’ areas of responsibility”(42,51)"lack of a vision in organizations on the NPs’ role"(42)  “lack of collaboration between different services and between health care professionals”(3) “lack of acceptance of local secondary care services or departments of nurse’s referral”(4,22,27,38,42,45,53)” consultant accepting NPs referrals did not communicate with NPs directly, the letter was addressed to the patient’s GP”(38)  ”lack of collegial and administrative support”(1,7,15,41,52) “administrators do not treat NPs and physicians equally or share information equally”(50) “lack of support, on-site visits by managers and more informal communication can lead a feeling to of being cut-off”(10)”NPs did not receive the same level of support as physicians did to deliver the same services”(16,26) “physicians have better access then NPs to organizational resources”(26) “lack of respect from other clinicians, support and administrative staff”(7) “physicians do not treat NPs as equal colleagues”(50)  “nurses low visibility”(26,50,51)  “professional isolation”(4,7,9,10,39,40,52)  *goal and feedback*  “lack of clear objectives/goals for nurse’s role development and success within the clinical setting or working environment”(7,9)  *compatibility*  “lack of recognition of the nurse role by others (3) resulting in either a duplication of services or reduced access to potential nursing services”(6)“Perceptions that the nurse role duplicates that of other professionals”(23,36)  *organizational incentives-contractual context*  “low wage as compared with other colleagues, lack of bonuses or raises in salary”(7,34)“NPs felt underpaid and undervalued by health care organization”(7,18) |
|  | E. Readiness for  implementation | *access to knowledge and information*  “NP access to PHU programs for their clients”(4) | *available resources*  “lack of resources”(3,11,23,35)”lack of appropriate patient-care equipment and infrastructure”(13,23,26,27,31) “lack of tools to understand the resource demand and manage staff allocation”(34) “electronic health records and billing codes did not adequately reflect NP practice”(48) “electronic patients records and computer operation are not ready to integrate APNs practice”(23) “under investment in information technology”(34) |
| **4. Individual**  **characteristics** |  |  |  |
|  | A. Knowledge & beliefs  about intervention | “health care providers education about the NP role”(24) “role clarity is an important step in gaining acceptance of collegial partners”(49)  *from GP’s perspective – facilitators to employ nurse’s role*  “GPs support among the staff and the collaboration with another practice”(44)”GPs previous experience of doctor-nurse collaboration”(1,24,38,44)  *from GP’s perspective – reason to employ*  “NPs view is complementary to the medical view of the GPs”(44,53) “NP would enhance the delivery and quality of healthcare(42,43) improving access to care (5) and increasing the total number of appointment available”(45)  *from GP’s perspective – changing role of GP*  “more coordinating role to handle complex cases (19,42) and expand their practice(43)”“reduction of doctor’s workload”(21,29,33,42)  *acceptability*  “primary care personnel satisfaction with NPs (8,19,39) or CMHN in their practice(11)”“GPs recognized the added value of APNs/NPs in primary care”(8,29,42) “Physicians acceptance of the NPs role(20) and the new model of partnership working(13)”  *From nurse’s perspective*  “Nurses perceived their work as being valuable and worthwhile because it is “different from” what other health professional provide(6), they had something additional to offer to patients(38)” “Nurses felt that they help provide better care and increase patient safety and satisfaction”(8,10,12,22,29,33,44,48) “Nurses perceptions that their work make a difference in clients’ health practices or health status”(1,6,12) | *acceptability*  “GPs’ confusion regarding the NP’s scope of practice and professional role boundaries”(43)“confusion regarding job titles”(54) “lack of a common understanding of the role”(1,4,29,39–41,50,55,6,7,13,15,19,20,22,26)“lack of knowledge regarding education and/or scope of practice”(3,5,7,11,19,21,25,27,44)  “stakeholders skepticism”(25,36) “poor GPs’ and other nurses’ acceptance of NPs”(43)”resistance and opposition by GPs”(23,24,42) “GPs reticence: there was no demonstration or evidence of a need to employ NPs to meet any major deficit in service provision(21)”  “GPs concern about workload, competition, fragmentation and duplication of services”(43,44) “GPs perceived a threat from NP role”(40)  ”physicians lack of confidence or trust in the nurses’ capabilities/competencies (3,13,17,19,20,40,43,50) “GPs perception that NPs were used as a cheap option by the Government”(43) “reluctance to consider NPs as an alternative workforce to GPs”(1) |
|  | B. Self-efficacy | *self confidence*  “Nurses were moderately confident in their skills”(55)“awareness of own limits”(8,29)  *prior work experience*  “nurse level of expertise, experience(3,8)”  *sense of meaning*  “Nurses felt a great sense of meaning for their work”(7,55) | *self confidence*  “self-doubt”(15,20)“nurses underestimated their competence”(51)“colleagues did not utilize NP as a resource”(49). |
|  | C. Individual stage of change |  | “opposition from own nursing profession”(23) “nurses’ unwillingness in taking on the increased responsibility inherent in the role.”(40) |
|  | D. Individual identification  with organization |  |  |
|  | E. Personal attributes | “personality and philosophy of the physicians”(4)  “nurse personal suitability to community work”(10)“nurse attribute: non-judgmental, honest, non-threatening.”(6) | “personality and philosophy of the physicians”(4) |
| **5. Process** |  |  |  |
|  | A. Planning | “project illustrating the potential of the nurse role”(23) “well planned integration, definition of roles and functions and involvement of the whole team”(23) “review nurse service”(36,41)  “appropriate workforce planning and training to replace (refer to demographic profile of nurses)”(34) | “lack of clarity or direction about reconfiguration of the role”(28)”uncertainty of role (as new services were adapted to meet changing needs”(10) |
|  | B. Engaging | “strategic commitment to NP development from employing organization (beginning from support to undertake postgraduate study through to employment as an NP)”(1) ”strategic alliance between NP and HA to role development and integration”(49)(48)“mentorship by policy leaders as a way to increase NPs leadership capacity”(48,49) “NP involvement in developing their role”(4) “nursing staff involvement in the drafting of job description”(54) “GPs engagement to help develop a new model in primary care”(29)  *external change agents*  “social service involvement”(11) Universities as driving force in supporting APN implementation role”(37) | “lack of leadership to guide the change”(32)“lack of nursing leadership”(1) “lack of engagement between Director of Nursing, of District Health Boards and PHC nurses”(1) “lack of NPs’ representation in important committees”(50) “lack of nurse involvement in the organizations”(26,41) |
|  | C. Executing | "support nursing staff in their professional development based on evidence-based practice"(3)“mentorship program”(56) “team building programs to team progression during the early stages of development”(30) “team building strategies”(48,51) “ negotiation of the nurse’s role and autonomy”(23,38,53) | “project implementation from top-down and lack of information”(23)“slow implementation process”(23)  “nurses felt difficult to identify any real change to their role or responsibilities”(13) |
|  | D. Reflecting & evaluating | “nurses’ need to evaluate their effectiveness”(6)“mechanism by which NPs subjected their practice to scrutiny: meetings with other nurses and NPs, contribution to the teaching of undergraduate NPs, research and audit, personal reflection on practice”(38) | “difficulties in identifying outcomes to measure,  given the nature of nurses work (immediate outcomes may  be less tangible, while more objective outcomes in terms  of illness prevention tend to be long-term)”(6)“lack of tools and resources to track and measure NP contributions: i.e. billing code used to track clinical services did not fully capture the holistic care provided to clients and community”(48)  “lack of knowledge about outcome measures”(41) “The audit process was restricted to a few areas of activity”(41)”  “lack of NPs performance feedback”(50) |

Legend: APN-Advanced Nurse Practitioner; CMHN-Community Mental Health Nurse; GP-General Practitioner; HA-Health Authority; NMC-Nursing and Midwifery Council; NP-Nurse Practitioner; PCP-Primary Care Provider; PHC-Primary Health Care; PHU-Public Health Unit; RN-Registered Nurse.

References

1. Adams S, Carryer J, Nz F. Establishing the nurse practitioner workforce in rural New Zealand : barriers and facilitators. J Prim Heal Care. 2019;11(2):152–9.

2. Athey EK, Leslie MS, Briggs LA, Park J, Falk NL, Pericak A, et al. How important are autonomy and work setting to nurse practitioners’ job satisfaction? J Am Assoc Nurse Pract. 2016 Jun 1;28(6):320–6.

3. Boman E, Glasberg A-L, Levy-Malmberg R, Fagerstrom L. “Thinking outside the box”: advanced geriatric nursing in primary health care in Scandinavia. BMC Nurs. 2019;18:25.

4. De Guzman A, Ciliska D, DiCenso A. Nurse practitioner role implementation in ontario public health units. Can J Public Heal. 2010;101(4):309–13.

5. Donelan K, DesRoches CM, Dittus RS, Buerhaus P. Perspectives of physicians and nurse practitioners on primary care practice. N Engl J Med. 2013 May 16;368(20):1898–906.

6. Reutter LI, Ford JS. Perceptions of public health nursing: Views from the field. J Adv Nurs. 1996 Jul;24(1):7–15.

7. Faraz A. Facilitators and barriers to the novice nurse practitioner workforce transition in primary care. J Am Assoc Nurse Pract. 2019;31(6):364–70.

8. Kraus E, DuBois JM. Knowing Your Limits: A Qualitative Study of Physician and Nurse Practitioner Perspectives on NP Independence in Primary Care. J Gen Intern Med. 2017;32(3):284–90.

9. Drury V, Francis K, Dulhunty G. The Lived Experience of Rural Mental Health Nurses. Online J Rural Nurs Heal Care. 2005;5(1):19–27.

10. Kipping CJ. Exploring mental health nurses’ expectations and experiences of working in the community. J Clin Nurs. 1998 Nov;7(6):531–8.

11. Crawford P, Carr J, Knight A, Chambers K, Nolan P. The value of community mental health nurses based in primary care teams: “Switching the light on in a cellar.” J Psychiatr Ment Health Nurs. 2001 Jun;8(3):213–20.

12. Carryer J, Adams S. Nurse practitioners as a solution to transformative and sustainable health services in primary health care: A qualitative exploratory study. Collegian. 2017 Dec 1;24(6):525–31.

13. Parfitt BA, Cornish F. Implementing Family Health Nursing in Tajikistan: from policy to practice in primary health care reform. Soc Sci Med. 2007;

14. Hansen-Turton T, Ware J, Bond L, Doria N, Cunningham P. Are managed care organizations in the United States impeding the delivery of primary care by nurse practitioners? A 2012 update on managed care organization credentialing and reimbursement practices. Popul Health Manag. 2013 Oct 1;16(5):306–9.

15. Carryer J, Boddy J, Budge C. Rural nurse to nurse practitioner: an ad hoc process. J Prim Health Care. 2011 Mar;3(1):23–8.

16. Weiland SA. Understanding nurse practitioner autonomy. J Am Assoc Nurse Pract. 2015 Feb 1;27(2):95–104.

17. Wilson A, Pearson D, Hassey A. Barriers to developing the nurse practitioner role in primary care - The GP perspective. Fam Pract. 2002 Dec;19(6):641–6.

18. Côté N, Freeman A, Jean E, Denis J-L. New understanding of primary health care nurse practitioner role optimisation: the dynamic relationship between the context and work meaning. BMC Health Serv Res. 2019 Nov;19(1):882.

19. Fletcher CE, Baker SJ, Copeland LA, Reeves PJ, Lowery JC. Nurse practitioners’ and physicians’ views of NPs as providers of primary care to Veterans: Health policy and systems. J Nurs Scholarsh. 2007 Dec;39(4):358–62.

20. Fletcher CE, Copeland LA, Lowery JC, Reeves PJ. Nurse practitioners as primary care providers within the VA. Mil Med. 2011;176(7):791–7.

21. Carr J, Armstrong S, Hancock B, Bethea J. GPs’ perceptions of the nurse practitioner role in primary care. Br J Community Nurs. 2002 Aug;7(8):408–13.

22. Gould ON, Johnstone D, Wasylkiw L. Nurse practitioners in Canada: beginnings, benefits, and barriers. J Am Acad Nurse Pract. 2007 Apr;19(4):165–71.

23. Jean E, Sevilla Guerra S, Contandriopoulos D, Perroux M, Kilpatrick K, Zabalegui A. Context and implementation of advanced nursing practice in two countries: An exploratory qualitative comparative study. Nurs Outlook. 2019 Jul 1;67(4):365–80.

24. Martin-Misener R, Reilly SM, Vollman AR. Defining the role of primary health care nurse practitioners in rural Nova Scotia. Can J Nurs Res. 2010 Jun;42(2):30–47.

25. Lindblad E, Hallman EB, Gillsjö C, Lindblad U, Fagerström L. Experiences of the new role of advanced practice nurses in Swedish primary health care-A qualitative study. Int J Nurs Pract. 2010 Feb;16(1):69–74.

26. Poghosyan L, Nannini A, Smaldone A, Clarke S, O’Rourke NC, Rosato BG, et al. Revisiting scope of practice facilitators and barriers for primary care nurse practitioners: a qualitative investigation. Policy Polit Nurs Pract. 2013 Feb;14(1):6–15.

27. Sullivan-Bentz M, Humbert J, Cragg B, Legault F, Laflamme C, Bailey PH, et al. Supporting primary health care nurse practitioners’ transition to practice. Can Fam Physician. 2010;56(11):1176–82.

28. Carr SM. Refocusing health visiting - Sharpening the vision and facilitating the process. J Nurs Manag. 2005 May;13(3):249–56.

29. Gysin S, Sottas B, Odermatt M, Essig S. Advanced practice nurses’ and general practitioners’ first experiences with introducing the advanced practice nurse role to Swiss primary care: A qualitative study. BMC Fam Pract. 2019 Nov 27;20(1).

30. Burke T, O’ Neill C. Community nurses working in piloted primary care teams: Irish Republic. Br J Community Nurs. 2010 Aug;15(8):398–404.

31. Rapport F, Maggs C. Measuring care : the case of district nursing. J Adv Nurs. 1997;25:673–80.

32. Domm E, Urban AM. Public health nurse perceptions of evolving work and how work is managed: A qualitative study. In: Journal of Nursing Management. Blackwell Publishing Ltd; 2020. p. 2017–24.

33. Ljungbeck B, Sjögren Forss K. Advanced nurse practitioners in municipal healthcare as a way to meet the growing healthcare needs of the frail elderly: A qualitative interview study with managers, doctors and specialist nurses. BMC Nurs. 2017 Nov 16;16(1).

34. Drennan VM. More care out of hospital? A qualitative exploration of the factors influencing the development of the district nursing workforce in England. J Health Serv Res Policy. 2019 Jan;24(1):11–8.

35. McCullough K, Whitehead L, Bayes S, Williams A, Cope V. The delivery of Primary Health Care in remote communities: A Grounded Theory study of the perspective of nurses. Int J Nurs Stud. 2020 Feb;102:103474.

36. Drennan V, Goodman C, Manthorpe J, Davies S, Scott C, Gage H, et al. Establishing new nursing roles : a case study of the English community matron initiative. 2011;2948–57.

37. Zug KE, Cassiani SHDB, Pulcini J, Bassalobre Garcia A, Aguirre-Boza F, Park J. Enfermagem de prática avançada na América Latina e no Caribe: Regulação, educação e prática. Rev Lat Am Enfermagem. 2016;24.

38. Price A, Williams A. Primary care nurse practitioners and the interface with secondary care : a qualitative study of referral practice. J Interprof Care. 2003;17(3):239–50.

39. Hunter KF, Murphy RS, Babb M, Vallee C. Benefits and Challenges Faced by a Nurse Practitioner Working in an Interprofessional Setting in Rural Alberta. Nurs Leadersh (Tor Ont). 2016;29(3):61–70.

40. Main R, Dunn N, Kendall K. “Crossing professional boundaries”: Barriers to the integration of nurse practitioners in primary care. Educ Prim Care. 2007;18(4):480–7.

41. Plews C, Billingham K, Rowe A. Public health nursing: Barriers and opportunities. Heal Soc Care Community. 2000;8(2):138–46.

42. Lovink MH, van Vught AJAH, Persoon A, Schoonhoven L, Koopmans RTCM, Laurant MGH. Skill mix change between general practitioners, nurse practitioners, physician assistants and nurses in primary healthcare for older people: a qualitative study. BMC Fam Pract. 2018 May;19(1):51.

43. Mackay B. General practitioners’ perceptions of the nurse practitioner role: an exploratory study. N Z Med J. 2003 Mar 14;116(1170):U356.

44. van der Biezen M, Derckx E, Wensing M, Laurant M. Factors influencing decision of general practitioners and managers to train and employ a nurse practitioner or physician assistant in primary care: a qualitative study. BMC Fam Pract. 2017 Feb;18(1):16.

45. Perry C, Thurston M, Killey M, Miller J. The nurse practitioner in primary care: alleviating problems of access? Br J Nurs. 2005;14(5):255–9.

46. Parker R, Forrest L, Ward N, McCracken J, Cox D, Derrett J. How acceptable are primary health care nurse practitioners to Australian consumers? Collegian. 2013;20(1):35–41.

47. Craswell A, Dwyer T. Reasons for choosing or refusing care from a nurse practitioner: Results from a national population-based survey. J Adv Nurs. 2019 Dec;75(12):3668–76.

48. Burgess J, Martin A, Senner W. A framework to assess nurse practitioner role integration in primary health care. Can J Nurs Res. 2011 Mar;43(1):22–40.

49. Burgess J, Purkis ME. The power and politics of collaboration in nurse practitioner role development. Nurs Inq. 2010 Dec;17(4):297–308.

50. Poghosyan L, Norful AA, Martsolf GR. Organizational structures and outcomes of newly hired and experienced nurse practitioners in New York State. Nurs Outlook. 2017 Sep;65(5):607–14.

51. Clancy A, Svensson T. Perceptions of public health nursing practice by municipal health officials in Norway. Public Health Nurs. 2009 Sep;26(5):412–20.

52. Conger M, Plager K. Advanced nursing practice in rural areas: connectedness versus disconnected...: EBSCOhost. Online J Rural Nurs Heal Care. 2008;8(1):24–38.

53. MacDonald JM. Higher level practice in community nursing: part two. Nurs Stand. 2005;20(10):41–9.

54. Dunt DR, Temple-Smith MJ, Johnson KA. Nursing outside hospitals: the working experience of community nurses: job characteristics. Int J Nurs Stud. 1991;28(1):27–37.

55. Faraz A. Novice nurse practitioner workforce transition and turnover intention in primary care. J Am Assoc Nurse Pract. 2017 Jan 1;29(1):26–34.

56. Jarrell L. Professional development and mentorship needs of nurse practitioners. J Nurses Prof Dev. 2016 Jan 1;32(1):26–32.
